# The Adjuvants Polyphosphazene (PCEP) and a Combination of Curdlan Plus Leptin Promote a Th17-Type Immune Response to an Intramuscular Vaccine in Mice

**DOI:** 10.3390/vaccines9050507

**Published:** 2021-05-14

**Authors:** Alyssa Chaffey, Glenn Hamonic, Dylan Chand, George K. Mutwiri, Heather L. Wilson

**Affiliations:** 1 Vaccinology & Immunotherapeutic Program, School of Public Health, University of Saskatchewan, 104 Clinic Place, Saskatoon, SK S7N 2Z4, Canada; alyssa.chaffey@usask.ca (A.C.); djc672@mail.usask.ca (D.C.); george.mutwiri@usask.ca (G.K.M.); 2 Vaccine & Infectious Disease Organization (VIDO), University of Saskatchewan, 120 Veterinary Road, Saskatoon, SK S7N 5E3, Canada; 3 Large Animal Sciences, Western College of Veterinary Medicine, University of Saskatchewan, 52 Campus Drive, Saskatoon, SK S7N 5B4, Canada; glenn.hamonic@usask.ca

**Keywords:** mice, ovalbumin, cell-mediated immunity, flow cytometry, interleukin-17, Th17-type T cells, polyphosphazene, curdlan, leptin

## Abstract

Our aim was to determine whether polyphosphazene (PCEP), Curdlan (β-glucan, a dectin-1 agonist), and Leptin could act as adjuvants to promote a Th17-type adaptive immune response in mice. Mice were vaccinated via the intramuscular route then boosted three weeks later with Ovalbumin plus: PCEP, Leptin, Curdlan, PCEP+Curdlan, Curdlan+Leptin, or saline. Mice vaccinated with OVA+PCEP and OVA+Curdlan+Leptin showed significantly higher frequency of antigen-specific CD4+ T cells secreting IL-17 relative to OVA-vaccinated mice. No formulation increased the frequency of CD4+ T cells secreting IL-4 or IFNγ. Since activation of innate immunity precedes the development of adaptive immunity, we wished to establish whether induction of Th17-type immunity could be predicted from in vitro experiments and/or from the local cytokine environment after immunization with adjuvants alone. Elevated IL-6 and TGFβ with reduced secretion of IL-12 is a cytokine milieu known to promote differentiation of Th17-type immunity. We injected the immunostimulants or saline buffer into murine thigh muscles and measured acute local cytokine production. PCEP induced significant production of IL-6 and reduced IL-12 production in muscle but it did not lead to elevated TGFβ production. Curdlan+Leptin injected into muscle induced significant production of TGFβ and IL-17 but not IL-6 or IL-12. We also stimulated splenocytes with media or PCEP, Leptin, Curdlan, PCEP+Curdlan, Curdlan+Leptin, PCEP+Leptin, and PCEP+Curdlan+Leptin and measured cytokine production. PCEP stimulation of splenocytes failed to induce significant production of IL-6, IL-12, TGFβ, or IL-17 and therefore ex vivo splenocyte stimulation failed to predict the increased frequency of Th17-type T cells in response to the vaccine. Curdlan-stimulated splenocytes produced Th1-type, inducing cytokine, IL-12. Curdlan+/-PCEP stimulated TGF-β production and Curdlan+Leptin+/- PCEP induced secretion of IL-17. We conclude that PCEP as well as Curdlan+Leptin are Th17-type vaccine adjuvants in mice but that cytokines produced in response to these adjuvants in muscle after injection or in ex vivo cultured splenocytes did not predict their role as a Th17-type adjuvant. Together, these data suggest that the cytokine environments induced by these immunostimulants did not predict induction of an antigen-specific Th17-type adaptive immune response. This is the first report of these adjuvants inducing a Th17-type adaptive immune response.

## 1. Introduction

Induction of appropriate co-stimulatory molecules on antigen-presenting cells facilitates the differentiation of CD4+ cells into helper T cell (Th) subtypes. CD4+ Th1 cells produce IFNγ, interleukin (IL)-2, and tumor necrosis factor (TNF), and induce macrophage activation and cell-mediated immunity whereas CD4+Th2-cells produce IL-4, -10, and -13, induce B cell activation, antibody production, and inhibit phagocyte function [1]. CD4+ Th17 cells produce interleukin (IL)-17A, IL-17F, and IL-22 which can contribute to inflammation and autoimmunity [2,3,4,5,6,7,8] as well as protective immunity to vaccines against bacteria such as *Klebsiella pneumonia*, *Bordetella pertussis*, *Mycobacterium tuberculosis*, *Pseudomonas aerginosa*, and *Haemophilus influenzae* [9,10,11,12,13]. These CD4+ Th17 cells also induce immune responses which can contribute to the clearance of fungi such as *Candida albicans* [14] and persistent viral infections [15]. Studies in mouse splenocytes and lymph node cells have shown that the presence of transforming growth factor-β (TGFβ) and interleukin (IL)-6 with IL-23 promotes Th17 differentiation and that IL-12 can inhibit Th17 differentiation [16,17,18]. Other studies showed that Cholera toxin can promote Th17-type response in mice, likely due to secretion of IL-6 and other Th17 polarizing factors [19]. We wanted to identify an adjuvant or combination of adjuvants that could promote a Th17-type antigen-specific immune response in mice in response to intramuscular vaccination. 

Polyphosphazenes (PCEP) are a class of water-soluble synthetic polymers comprised of nitrogen atoms alternating with phosphorus atoms, the latter of which has organic side chains attached. PCEP is a powerful adjuvant that does not directly stimulate TLRs [20,21,22,23] but which likely mediates its activity through activation of the inflammasome and strong activation of the innate immune response [24]. We previously determined that intramuscular (i.m.) injection of mice with 50 μg PCEP as an immunostimulant (i.e., without an antigen and therefore independent of its function as a vaccine adjuvant) induced significant recruitment of neutrophils, macrophages, monocytes, dendritic cells (DCs), and lymphocytes to the site of injection [25]. Further, i.m. administration of 50 μg PCEP upregulated local gene expression of the inflammasome receptor, NLRP3, and it triggered local, rapid, and sustained (for at least 96 h) upregulated gene expression of IL-1β and IL-18, genes whose proteins are activated by the inflammasome. PCEP also showed local, rapid, and sustained upregulated gene expression of IL-2 and IFNγ (cytokines known to promote Th1-type immunity), IL-17 (cytokine associated with Th17-type immunity), and IL-4 and IL-13 cytokines (cytokines associated with induction of Th2-type immunity) [23]. Mice immunized with a vaccine consisting of 10 μg OVA plus 50 μg PCEP responded with induced recall production of IFNγ by splenic CD8+ and CD4+ T cells as well as induced significant but low-level production of the B cell activating cytokine IL-6, which did not lead to increased proliferation of enriched B cells [26]. In comparison, mice immunized s.c. with 5 μg X:31 influenza antigen plus 50 μg PCEP responded with an approximately equal number of IL-4 or IFNγ producing T cells in splenocyte population, indicating an antigen-specific mixed Th1 and Th2-type immune response and IL-6 gene expression showing a very strong peak after 24 hrs that then sharply declined [22]. Therefore, it was not clear whether PCEP directed a specific Th-type immune response. We intended to assess whether intramuscular vaccination with PCEP as an adjuvant induced Th17-type cell-mediated immune response (CMI).

C-type lectin receptors (CLRs) bind to pathogen-specific carbohydrate residues and are predominantly expressed in myeloid cells [27,28]. Dectin-1 is a CLR that recognizes β-glucans carbohydrates expressed by bacteria and fungi [29]. In an experiment that assessed TLR2 and Dectin-1 signaling in mouse hematopoietic stem and progenitor cells, it was determined that recognition of these β-glucans led to increased expression of MHCII, co-stimulatory molecules, cytokines, and ultimately an enhanced Th17 response [30]. Murine RAW264.7 macrophages stimulated with β-glucan Curdlan-sulphate induced significant production of TNFα, IL-6, and IL-1β [31]. In mouse-bone-marrow-derived DCs stimulated with Curdlan, the cells responded with induced production of IL-2, IL-10, IL-6, and TNFα with a bias towards production of IL-23 rather than IL-12 [32]. Similarly, human monocyte-derived DCs cultured in the presence of Curdlan showed induced secretion of IL-23, IL-1β, IL-6, and low levels of IL-12p70 as determined by ELISA [33]. Importantly, Curdlan-activated murine and human DCs promoted the polarization of naïve T cells towards Th17-type immunity [32,33]. In a recent study, Curdlan was observed to elicit a Th17 response when administered with PLGA-encapsulated *Pseudomonas aerginosa* PopB vaccine antigen, where it conferred protection against experimental acute pneumonia [34]. For these reasons, we investigated whether Curdlan could act to induce a Th17-type immune response.

Leptin is a protein that regulates cellular homeostasis and glycemic control as well as acts as an inflammatory molecule capable of activating the innate and adaptive arms of the immune system [35,36,37]. The Leptin receptor is expressed on the surface of peripheral and bone-marrow-derived immune cells as well as in major organs and muscle [38,39,40,41,42]. Leptin promotes the activation of natural killer cells (NK), neutrophils, granulocyte chemotaxis, and the secretion of TNFα, IL-6, and IL-12 from macrophages during induction of the innate immune response [37,42,43]. However, studies in mice showed that Leptin signaling may be critically required for Th17 differentiation and T cell survival [37,44,45], which makes it a potential adjuvant for Th17 directed cell-mediated immunity (CMI). 

Subunit vaccines have a long track record of safety but are often poorly immunogenic and require adjuvants to direct and/or enhance the adaptive immune response. We investigated whether intramuscular immunization of mice with ovalbumin formulated with PCEP, Curdlan, and Leptin as adjuvants induced Th17 CMI. Next, we investigated whether the cytokine environment induced in the muscle 48 h after injection with the immunostimulants (in the absence of antigen) and their effect on cytokine production on primary murine splenocytes was predictive of the observed T-cell-mediated vaccine-specific adaptive immune response.

## 2. Materials and Methods

The majority of these methods (as well as results and discussion) are previously described in the thesis by Chaffey, University of Saskatchewan (Chaffey, A. Identification of Immunostimulatory Adjuvant(s) That Will Promote a Th17-Type of Immune Response (thesis); University of Saskatchewan. Available online: https://harvest.usask.ca/bitstream/handle/10388/7959/CHAFFEY-THESIS-2017.pdf?sequence=1&isAllowed=y (accessed on 2 February 2021) [46].

### 2.1. Animal Experiments

All animal experiments were conducted according to the Guidelines for the Care and Use of Laboratory Animals as indicated by the Canadian Council on Animal Care and was approved by the Animal Care Committee of the University of Saskatchewan; 199402012, Dec 2016.

#### Mouse Immunization, Intramuscular Injections, and ex vivo Splenocytes Stimulations

**Figure 1:**
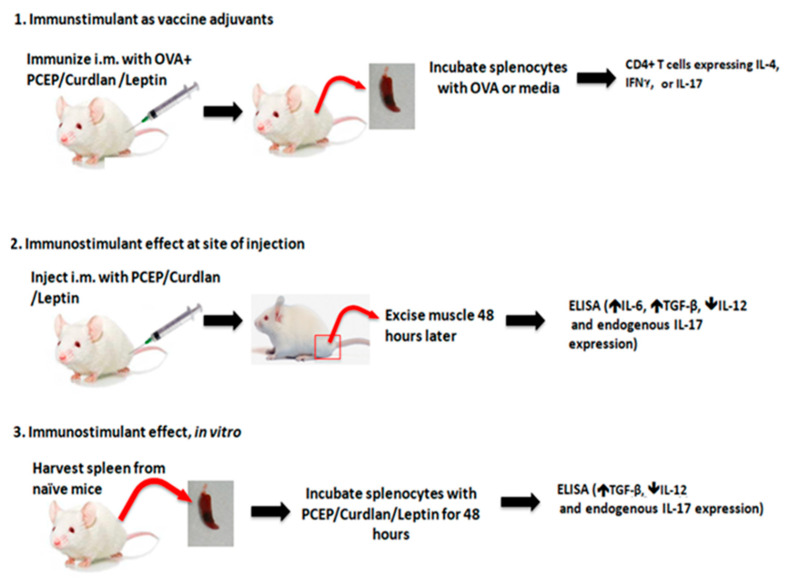
Schematic of experimentation: 1. Mice were immunized via the intramuscular route with OVA plus adjuvants, then splenocytes were excised and recall cell-mediated immune response was measured by cytokine production and an increase in CD4+ T cells expressing IL-4, IL-17, or IFNγ. 2. Mice were injected with immunostimulants into the thigh muscle. Two days later, muscles were isolated and production of IL-6, IL-12, IL-17, and TGFβ was measured. 3. Spleens from naïve mice were isolated then stimulated with the adjuvant(s) ex vivo for 48 h. The cytokines produced in the supernatants were measure by ELISA.

Six- to eight-week old BALB/c mice were obtained one week prior to experimentation to allow them to acclimatize, then they were randomly assigned to groups. Figure 1 lays out the basic outline of experiments performed on vaccinated mice (1), on mice injected i.m. with the immunostimulants (2), and ex vivo splenocytes from naïve mice response to immunostimulants (3). Dose response assays with the splenocytes showed that 2.5 μg PCEP was optimal for cytokine production. Because we wanted to assess whether i.m. immunization and/or splenocyte stimulation triggered an environment predictive of IL-17 T cell vaccine response, we opted to perform the vaccine trial at this low dose.

### 2.2. Vaccine Trial

Mice (*n* = 8 per group) were immunized into the thigh muscles with 50 μL total volume (25 μL per thigh) with OVA (0.5 μg/total dose), poly[di(sodium carboxylatoethylphenoxy)phosphazene] (PCEP, 2.5 μg/dose, Idaho National Laboratory, Idaho Falls, ID, US), Curdlan (0.5 μg/total dose, Wako Pure Chemical Industry, Ltd. Japan)), Leptin (0.5 μg/dose, Millipore Sigma, Oakville, Ontario, Canada), PCEP+Curdlan, Curdlan+Leptin, PCEP+Curdlan+Leptin or phosphate-buffered saline (PBS) on day 0 and day 21 (booster). Vaccine diluent was PBS, pH 7.4 (Gibco, Life Technologies). Spleens were excised on day 35 and processed to a single-cell suspension. Cells were stimulated with OVA (1 μg/mL) or left unstimulated for 48 h then subjected to flow cytometric analysis to immunotype the T cell population that were induced in response to OVA. 

Local cytokine production after intramuscular injection with immunostimulants: mice were injected with 50 μL total volume (25 μL per thigh) with PCEP (2.5 μg/dose), Curdlan (0.5 μg/dose), Leptin (0.5 μg/dose), PCEP+Curdlan, Curdlan+Leptin, or PBS into the thigh muscles. After 48 h, muscles were excised, processed into a single cell suspension, and cells were lysed (details below). 

### 2.3. Ex Vivo Response to Immunostimulants

Splenocyte from naive mice (*n* = 6) were isolated, processed into a single cell suspension, then cultured with PCEP (25 μg/mL), Curdlan (1 μg/mL), Leptin (1 μg/mL), PCEP+Curdlan, Curdlan+Leptin, or PBS for 48 h. IL-6, IL-12, TGFβ, and IL-17 cytokine production was measured by ELISA. 

#### Isolation of Muscle Tissue

The muscle tissue from both injection sites was excised 48 h later and placed into a 2 mL micro tube (VWR, Edmonton, AB, Canada) with 2.4 mm zirconia beads (Biospec Products, Inc., Bartlesville, OK, USA). The tissues were homogenized in a MINI Bead Beater (Biospec Products, Inc., Bartlesville, OK, USA) at 4800 osc/sec for 10 s to disrupt the tissues. The cells were frozen at −20 °C and thawed at 37 °C to lyse remaining cells. The homogenate were subjected to centrifugation (Sorvall Legend RT, Mandel) at 10,000× *g* for 5 min and the supernatant was removed and frozen at −20 °C for ELISA analysis. Supernatants were subjected to IL-6, IL-12, TGFβ, and IL-17 cytokine analysis.

### 2.4. Isolation of Splenocytes

Spleens were isolated and processed as detailed in Baca-Estrada et al., (1996) [47].

### 2.5. Cytokine Detection by ELISA in Mice

For ELISA analysis, R&D Systems Duoset kits (Fisher Scientific, Pittsburgh, PA, USA) were used to quantify IL-4, IL-6, IL-12, IL-17, TGFβ, and IFNγ as per the manufacturer’s protocol. All assays were performed on Immulon 2, 96-well microtiter plates (Dynex Technology Inc., Chantilly, VA, USA). Limit of detection for each cytokine in each experiment is indicated in the figure legend. Antibodies are detailed in Table 1.

### 2.6. Splenocyte T cells Immunotyping Using Intracellular Flow Cytometry

Splenocytes (100 μL of 1.0 × 10^6^ cells/well) from vaccinated mice were added to 96-well tissue culture plates and restimulated with either 100 μL of AIM V and 10% FBS alone (media control) or with 10 μg/mL OVA for 48 h. Next, 20 μL of Monensin (BD Bioscience, Franklin Lakes, NJ, USA) was added to each well for 8–12 h to prevent cytokine secretion from the cells. Cells were centrifuged at 500× *g* for 4 min at room temperature (RT), then the supernatant was removed by flicking the plates. Cells were washed twice with 200 μL flow cytometry (FCM) buffer (1.5 g sodium azide and 10.0 g Gelatin into 5 L PBS, pH 7.3) after which they were centrifuged at 500× *g* for 4 min at RT. Fix/Perm Solution (100 μL, BD Bioscience) was added to each well and all plates were incubated for a minimum of 30 min at 4 °C. Cells were centrifuged at 500× *g* for 4 min at RT and washed twice with Perm/Wash (BD Bioscience) buffer. Supernatants were removed by flicking the inverted plates, then cells were resuspended in 10 μL of pre-diluted antibody added to each well. Single stains, fluorochrome minus one (FMO), isotype, and positive and negative controls were used in this analysis and were added to wells at the time the antibody cocktails were added (See Table 2). The plates were incubated for 10 min at room temperature, then cells were washed with 200 μL Fix/Perm wash buffer. Cells were centrifuged at 500× *g* for 4 min at RT and resuspended in 200 μL Fix/Perm wash buffer. Cells were measured on FacsCalibur (BD Biosciences Bio-Rad Laboratories) and CellQuestPro by acquiring 100,000 events/sample. Data were analyzed on Kaluza (Beckman Coulter Life Sciences) and Graphpad prism. Examples of the gating strategy for splenocytes alone stained with antibodies for IL-4, IL-17, and IFNγ and gating for cells from vaccinated mice mock-stimulated or stimulated with OVA and stained with these cytokine antibodies are shown in Supplementary Figures S1 and S2, respectively.

### 2.7. Statistical Analyses

Statistical analyses were carried out using Graph-Pad Prism 6 software (GraphPad Software, San Diego, CA, USA). Differences in the cytokine production were identified using a non-parametric Kruskal–Wallis ANOVA test where Dunn’s multiple comparisons test was used post hoc to identify statistically significant differences in cytokine production. Differences in the frequency of OVA-stimulated or unstimulated CD4+ T cells were determined using Wilcoxon t tests and differences across treatments were determined using Kruskal–Wallis ANOVA as above. Differences were considered statistically significant at *p* < 0.05 (*), *p* < 0.01 (**), *p* < 0.001 (***), and *p* < 0.0001 (****) as stated in the text.

## 3. Results

### 3.1. CMI Response to Intramuscular OVA Vaccine Formulated with PCEP, Curdlan, and Leptin Adjuvants

**Figure 2:**
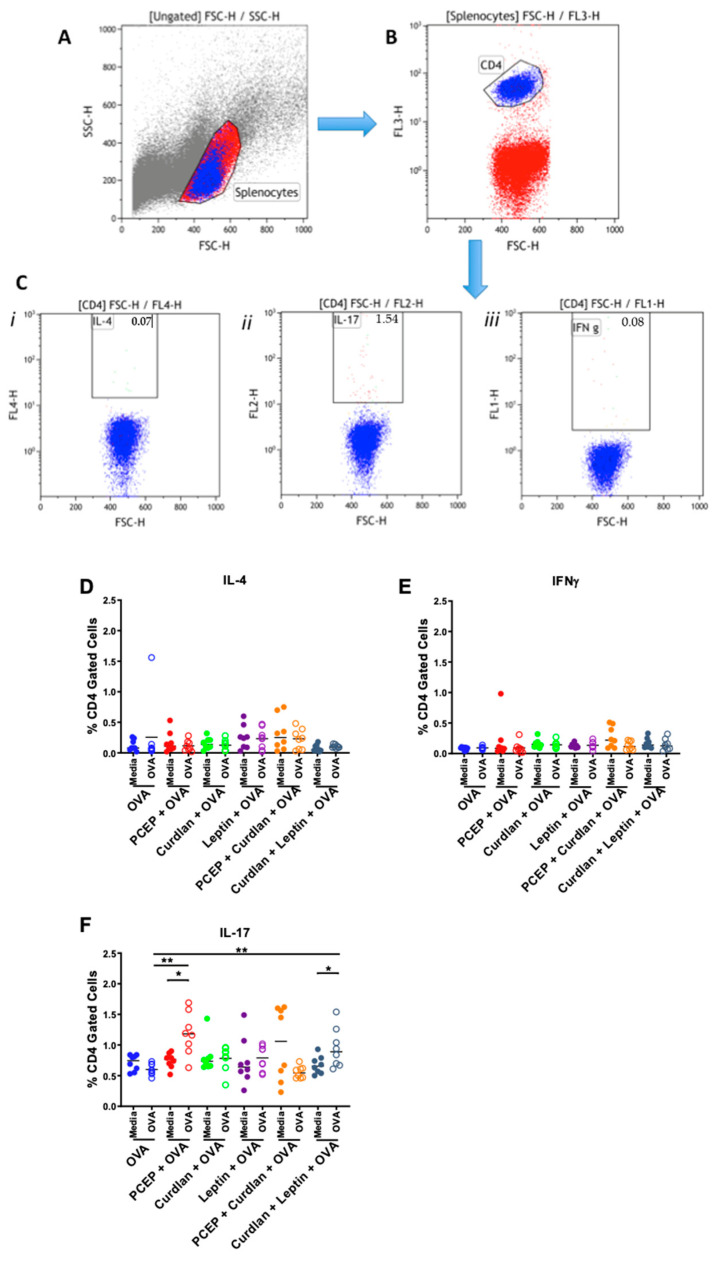
Vaccination with OVA plus PCEP or OVA+Curdlan+Leptin increased the frequency of CD4+IL-17+ T cells in splenocytes. BALB/c mice were mock-immunized or immunized with 50 μL (25 μL per leg) of OVA with or without the following adjuvants: PCEP (50 μg/mL)+OVA, Curdlan (10 μg/mL)+OVA, Leptin (10 μg/mL)+OVA, PCEP+Curdlan+OVA, and Curdlan+Leptin+OVA. Booster immunizations were administered on Day 21. Mice were euthanized and spleens were harvested on Day 35 and splenocytes were mock-stimulated with media or restimulated with OVA (1 μg/mL) and incubated for 48 h. Intracellular flow cytometric gating strategy: FCM was used to identify quantities of different T cell subsets within a spleen cell population from immunized BALB/c after 48 h incubation with OVA. (**A**) Portrays the live primary splenocyte gating from total population. (**B**) Portrays the gating of CD4+ cells from within the splenocyte gate. (**C**) Portrays gating of intracellular cytokines i: IL-4, ii: IL-17, and iii: IFNγ with the CD4+ gate. Data for A–C are from OVA-stimulated splenocytes from a representative mouse immunized with Curdlan+Leptin+OVA. Splenocytes were then stained with antibodies for CD4+ markers as well as (**D**) IL-4, (**E**) IFNγ, and (**F**) IL-17 to determine the frequency of CD4 cells with Th2-, Th1-, or Th17-type response. Statistical analysis was performed by one-way ANOVA and the differences between the OVA-stimulated cells were compared by Kruskal–Wallis and Dunn’s multiple-comparison tests, where ** *p* < 0.01. The OVA-stimulated cells were compared to matched unstimulated cells using Wilcox matched pairs signed rank test, where * *p* < 0.05, ** *p* < 0.01.

We wanted to ascertain whether PCEP, Curdlan, and Leptin adjuvants induced an OVA-specific Th-subtype cell-mediated immune response when used in a mouse i.m. vaccine. Mice were immunized in both thighs on day 0 and day 21 with OVA+PCEP, OVA+Curdlan, OVA+Leptin, OVA+PCEP+Curdlan, OVA+Curdlan+Leptin. Spleens were processed into a single-cell suspension 35 days after the primary immunization and stimulated with OVA or mock-stimulated for 48 h to allow for a recall response. Flow cytometric analysis was used to quantify different T cell subsets within a spleen cell population. Figure 2A portrays the live splenocyte population from total cellular population identified using side scatter (SSC-H) and forward scatter (FSC-H) and the smaller gate in Figure 2B indicates the CD4+ T cell population. Figure 2C portrays gating of intracellular cytokines (i) IL-4, (ii) IL-17, and (iii) IFNγ within the CD4+ gate. Data shown were obtained from a representative animal. We measured the frequency of splenic CD4+ T cells that were also positive for IL-4 (Figure 2D), IFNγ (Figure 2E), or IL-17 (Figure 2F) as a measure of Th2-, Th1-, or Th17-type immunity, respectively. Results indicated that no vaccine formulation increased the frequency of splenic CD4+IL-4+ Th2-type cells (Figure 2D) or CD4+IFNγ+ Th1-type cells (Figure 2E). However, splenocytes from mice vaccinated with PCEP+OVA (*p* < 0.01) or Curdlan+Leptin+OVA (*p* < 0.05) showed significantly increased frequency of CD4+IL-17+ T cells when cells were restimulated with OVA relative to mock-stimulated cells (*p* > 0.01; Figure 2F). Further, splenocytes from mice vaccinated with PCEP+OVA and OVA+Curdlan+Leptin showed significantly increased frequency of OVA-specific CD4+IL-17+ T cells relative to the mice vaccinated with OVA alone (*p* < 0.01). Thus, mice vaccinated with OVA+PCEP and OVA+Curdlan+Leptin showed a significant increase in the frequency of Th17-type CMI after vaccination.

Next, we wanted to determine whether injection of these adjuvants triggered induced expression of IL-6 and TGFβ and reduced IL-12, which may have led to the observed Th17-type immune response.

### 3.2. Local Cytokine Production in Response to PCEP, Curdlan, and/or Leptin Injected into Mouse Muscle

**Figure 3:**
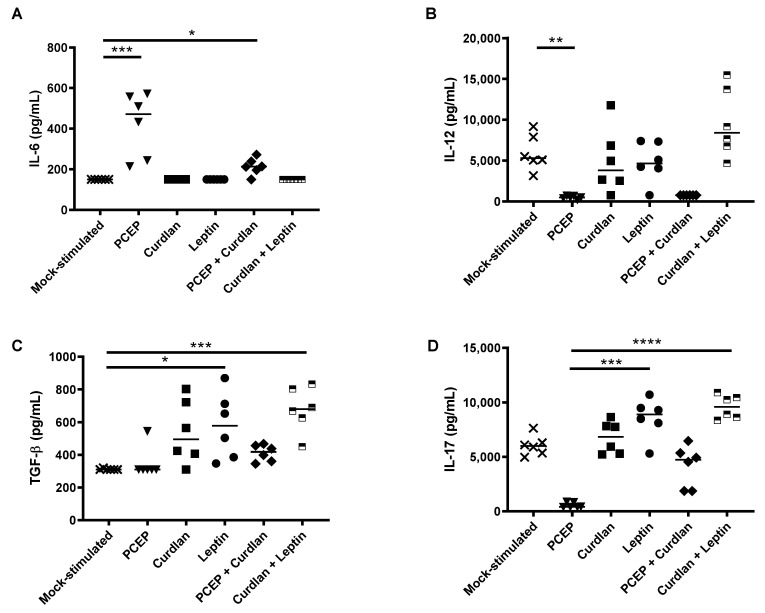
Cytokine expression in murine muscle cells harvested after injection. BALB/c mice were mock-injected with saline or injected (25 μL per thigh) with PCEP (50 μg/mL), Curdlan (10 μg/mL), Leptin (10 μg/mL), PCEP+Curdlan, and Curdlan+Leptin. Muscle cells were excised and homogenated 48 h post injection and cytokine concentration was analyzed by ELISA for (**A**) IL-6, (**B**) TGFβ, (**C**) IL-12, and (**D**) IL-17 (pg/mL). Values were calculated with respect to the ELISA Table 150. 310, 3120, and 1870 pg/mL, respectively. Data shown are presented as the mean of duplicate titres for individual biological replicates and the horizontal line represents the median value for the group. Statistical analysis was performed by one-way ANOVA and the differences between the treatments were compared by Kruskal–Wallis and Dunn’s multiple-comparison tests. Significant differences are depicted as follows: * *p* < 0.05, ** *p* < 0.01, *** *p* < 0.001, **** *p* < 0.0001.

To assess whether injection of PCEP, Curdlan, Leptin, PCEP+Curdlan, and Curdlan+Leptin into murine thigh muscles led to a change in cytokine profile, we performed ELISA analysis on muscle tissue 48 h post injection. Murine muscle tissue injected with PCEP and PCEP+Curdlan showed significantly higher local production of IL-6 relative to the cytokine levels in muscles obtained from naïve mice (Figure 3A; *p* < 0.001 and *p* < 0.05, respectively). Murine muscles injected with PCEP had significantly lower levels of IL-12 relative to the muscle from naïve mice (Figure 3B; *p* < 0.05). When measuring TGFβ production, muscle from mice injected with Leptin and Curlan+Leptin had significantly higher TGFβ relative to muscle from naïve mice (Figure 3C, *p* < 0.05 and *p* < 0.001, respectively). Finally, none of the immunostimulants triggered significantly different production of IL-17 in muscles relative to that observed in naïve mice (Figure 3D), although injection of PCEP resulted in significantly less IL-17 in the local environment relative to mice injected with Leptin (*p* < 0.001) or Curdlan+Leptin (*p* < 0.0001). Muscle cells from mice injected with PCEP showed a trend towards reduced IL-17 expression relative to the naïve mice but the results were not statistically significant (*p* < 0.16). Collectively, these data indicate that no immunostimulants injected intramuscularly led to increased IL-6, TGFβ, along with reduced IL-12, which is predicted to promote Th17-type immune cell differentiation.

Finally, we investigated whether splenocytes from naïve mice cultured with the immunostimulants promotes a cytokine environment conducive to Th17 cell differentiation.

### 3.3. Primary Splenocyte Cytokine Response to Acute Immunostimulants Exposure

**Figure 4:**
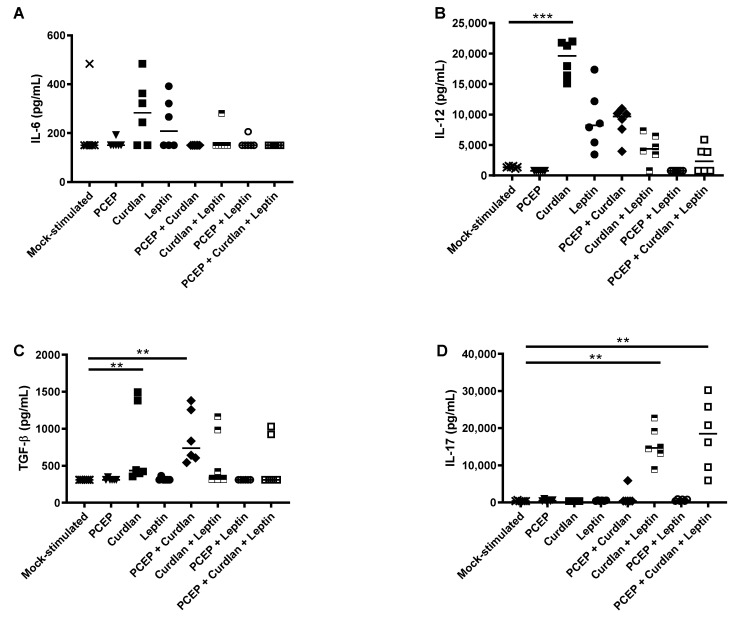
Expression of IL-6, TGF-β, IL-12 and IL-17 in murine splenocytes in response to immunostimulants, in vitro. Splenocytes were harvested from BALB/c mice (*n* = 6) and stimulated with PCEP (50 μg/mL), Curdlan (1 μg/mL), Leptin (1 μg/mL), PCEP+Curdlan, PCEP+Leptin, Curdlan+Leptin, and PCEP+Curdlan+Leptin. Supernatants were collected 48 h after treatment and analyzed by ELISA in pg/mL. Values were calculated with respect to the ELISA threshold limit of detection as (**A**) IL-6 (150), (**B**) TGFβ (370), (**C**) IL-12 (3120), (**D**) IL-17 (310) pg/mL. Data shown are presented as the mean of duplicate titres for individual biological replicates and the horizontal line represents the median value for the group. Statistical analysis was performed by one-way ANOVA and the differences between the treatments were compared by Kruskal–Wallis and Dunn’s multiple-comparison tests. Significant differences are depicted as follows: ** *p* < 0.01, *** *p* < 0.001.

Naïve splenocytes were cultured for 48 h with PCEP, Curdlan, Leptin, PCEP+Curdlan, and Curdlan+Leptin. No immunostimulants triggered increased secretion of IL-6 (Figure 4A). IL-12 production was significantly induced in splenocytes stimulated with Curdlan (*p* < 0.001) relative to mock-stimulated cells (Figure 4B). Curdlan (*p* < 0.01) and PCEP+Curdlan (*p* < 0.01) induced significantly more TGFβ relative to the mock-stimulated cells (Figure 4C). Finally, we observed that splenocytes stimulated with Curdlan+Leptin+/- PCEP had significantly induced secretion of IL-17 relative to mock-stimulated cells (Figure 4D; *p* < 0.01, *p* < 0.01). It may be that Curdlan is inducing a Th1-type environment, as it significantly induced expression of IL-12, a prominent inducer of this CD4+ subtype. These data indicate that none of the immunostimulants, alone or in combination, promoted an environment predictive to be conducive to induction of OVA-specific Th17 cell-type CMI. 

## 4. Discussion

Assessing the CMI response to vaccines in small animals requires euthanizing them and studying the antigen-specific induction of Th1-, Th2-, and Th17-type cell frequency or cytokine profile in the splenocyte population. We sought to determine whether the cell-mediated immune response to adjuvants, specifically adjuvants that may promote Th17-type immunity, could be predicted by studying the cytokine profile at the site of injection in the acute period and/or by studying the cytokine profile of primary splenocytes to immunostimulants.

Others have shown that Th17 differentiation of murine splenocytes requires the presence of TGFβ and IL-6 with IL-23 augmenting this response or with low-level IL-12 [16,17,18]. Cholera toxin is also known to induce IL-17-producing CD4+ Th17 cells in mice, possibly due to induction of IL-6 and IL-22, but not IFNγ, IL-5, IL-10, or IL-21, and that the toxin mediates its effects on the dendritic cells [19]. We determined that relative to the control mice, mice vaccinated with 0.5 μg PCEP+0.5 μg OVA showed a significant increase in the frequency of OVA-specific IL-17+CD4+ T cells (Figure 2). It may be that there is a dose-specific response, because our research has also shown that splenocytes from mice immunized subcutaneously with 5 μg influenza antigens formulated with 50 μg PCEP showed significant induction of IFNγ-secreting cells relative to the mice immunized with antigens alone [22]. The same dose of PCEP induced OVA-specific IFNγ production by both splenic CD8+ and CD4+ T cells as well as induced IL-6 production but not B cell proliferation [26]. As an immunostimulant, 100 μg PCEP injected intradermally in pigs showed elevated IL-6 after 96 h in response to PCEP [48]. We have also observed that 50 μg PCEP as an immunostimulant induced significant recruitment of neutrophils, macrophages, monocytes, dendritic cells (DCs), and lymphocytes at the site of injection as well as in the draining lymph nodes [25].

Because we observed significantly increased frequency of OVA-specific CD4+ Th17 T cells in the spleens of OVA+PCEP and OVA+Curdlan+Leptin-vaccinated mice, we would have predicted that primary murine splenocytes would respond to PCEP or Curdlan+Leptin with increased expression of IL-6, TGFβ, as well as decreased IL-12. Instead, primary splenocytes stimulated with PCEP failed to induce expression of IL-6, TGFβ, IL-12, or IL-17. The local cytokine response in the muscle in the acute period after injection showed that IL-12 was significantly reduced relative to naïve mice, which may indicate inhibited differentiation of Th1-type immune cells in the longer term. It also showed that PCEP in the muscle led to elevated secretion of IL-6 but not TGFβ (Figure 3). These data conflict with results from Awate et al. (2012) wherein they observed that 50 μg PCEP injected into murine muscle induced significant production of IL-6 and IL-12 after 48 h [23]; the reason for these differences is not known but may be a dose-response. Likewise, primary splenocytes stimulated with Curdlan+Leptin(+/-PCEP) failed to respond with increased IL-6, TGFβ, or decreased IL-12, although they did response with increased splenic IL-17 production. Injection of Curdlan+Leptin into muscles showed significantly higher production of TGFβ and IL-17 (and a trend towards increased production of IL-12), but not IL-6. The effect of the adjuvant combination Curdlan+Leptin+PCEP on OVA-specific IL-17 production in vaccinated mice should be assessed in future trials. Together, these data suggest that cytokines produced by muscle cells in response to immunostimulants are very different between the cytokines induced by splenocytes incubated with immunostimulants, ex vivo, and the OVA-specific response to PCEP or Curdlan+Leptin formulated vaccines over time.

Acute IL-17 production in muscle and ex vivo splenocytes suggests that Curdlan+Leptin(+/-PCEP) triggered IL-17 production by innate immune cells. Because we did not directly investigate what cells were responsible for the expression of IL-17, we can only speculate. The cells may have been memory Th17-cells, and this could have been clarified if we performed flow cytometric analysis and measured surface expression of CD4+, CCR6+, and CCR4+ [49]. Others have shown that neutrophils from cultured murine lungs produced IL-17A in response to Dectin-1 signaling [50], so it is possible that Curdlan signaling through Dectin-1 receptor induced expression of IL-17 from spleen neutrophils. However, because Dectin-1 receptor is also present on macrophages/monocytes and DCs, Curdlan could have activated these innate immune cells, which in turn induced activation of memory Th17 T cells [32,33,51,52]. Human DCs stimulated by Dectin-1 agonists have been shown to induce secretion of IL1β, IL-6, and IL-23, with low levels of IL-12, which primes naïve CD4 cells to differentiate into Th17 and Th1 cells [33]. Our results showed that Curdlan-stimulated splenocytes produced Th1-type, inducing cytokine IL-12, but this was not reflected in the OVA+Curdlan vaccinated mice, which did not show induction of IFNγ. Curdlan+Leptin(+/-PCEP)-stimulated splenocytes did not induce significantly more IL-12 than the mock-stimulated cells. Like Dectin-1 receptors, Leptin receptors are also present on neutrophils, monocytes, and lymphocytes, but Leptin signaling may be critically required for Th1 or Th17 differentiation [37,38,44]. Leptin induces functional and morphological changes in human dendritic cells (DCs), directing them towards Th1 priming and promoting DC survival [37,53]. Further, mouse CD4+ and CD8+ T lymphocytes express the Leptin receptor and respond to Leptin with promotion of T cell number and activation [54]. The Leptin receptor is highly expressed on the cell surface of human Tregs and acts as a negative signal for their proliferation [55]. Leptin inhibits Treg production and stimulates proliferation of naïve T cells into Th1 or Th17 cells [56] through expression of RORγt (ROR nuclear hormone receptor family), a transcription factor, and STAT3, both regulators of Th17 [41]. Thus, Curdlan and Leptin may act on the murine splenic Th17 cells directly or indirectly through induced innate immune cell activation.

There are some areas which would warrant further research and exploration as it pertains to the cytokines which indicate a Th17 response. Recent studies have indicated that along with TGFβ and IL-6 as necessary cytokines to induce a Th17 response, Activin and IL-21 can also induce Th17 differentiation of T cells in the absence of TGFβ [57,58,59]. In particular, activin, a member of the TGFβ family, is readily available in physiological conditions and thus may be a contributing factor to the differentiation of Th17 cells in the presence of IL-6 alone [57]. In assessment of the importance of the Ski-Smad4 axis in T cells, activin was able to induce signaling pathways similar to those activated by TGFβ, degrading Ski in activated T cells and promoting Th17 differentiation [55]. Naïve CD4+ T cells exposed to activin, IL-1β, IL-6, and IL-23 induced Th17 differentiation in vitro [60] at a relatively low level [61]. However, the mechanisms surrounding Th17 differentiation without TGFβ signaling still continue to be understood. Continued research of cytokines IL-23, IL-21, Activin, and the Ski-Smad4 axis in response to PCEP adjuvanted vaccines would be a valid future application of this experiment.

These experiments were focused on antigen-specific Th17 responses to OVA, so IL-17 production from other potential sources such as γδT, NK, iNKT, and neutrophils were not characterized. While CD4+ T cells are certainly not the only source of IL-17 cytokine and Th17 responses, the focus on antigen specific responses is consistent with the experiments and data assessed. Most experiments pertaining to PCEP often assess the adjuvant’s efficacy at recruiting cells to the site of infection and lymphatics system where antigen-specific responses typically take place [25,26]. Future considerations could certainly look at cell recruitment to the ability of PCEP or Curdlan+Leptin to induce lymphocyte traffic to the spleen as well as the immune response elicited.

## 5. Conclusions

Collectively, these data indicate that the adjuvants PCEP, as well as Curdlan+Leptin combination, are Th17-inducing adjuvants in mice. However, the local innate immune response induced by PCEP as well as Curdlan+Leptin as an immunostimulant at the site of injection and in splenocytes is not predictive of antigen-specific Th17-type immune cell differentiation.

## Figures and Tables

**Table 1:** Antibody targets, names, isotypes, and concentrations.

Target	Name of Antibody	Isotype	Concentration
CD4	PerCP-Cy™5.5 labeled Rat Anti-Mouse CD4	Rat (DA) IgG2a, κ	0.0625 μg/test
CD8	PE labeled Rat Anti-Mouse CD8b.2	Rat (LOU) IgG1, κ	0.25 μg/test
IL-4	APC labeled Rat Anti-Mouse IL-4	Rat IgG1	0.25 μg/test
IL-17	PE labeled Rat Anti-Mouse IL-17A	Rat IgG1, κ	0.25 μg/test
IFNγ	FITC labeled Rat Anti-Mouse IFNγ	Rat IgG1, κ	0.25 μg/test

**Table 2:** Flow cytometry fluorochrome cocktails. All antibodies were rat anti-mouse with a directly conjugated fluorochrome (BD Pharmingen).

Cocktail	Antibodies and Fluorochrome
Cocktail 1 (C1)	PerCP-Cy™5.5 labeled Rat Anti-Mouse CD4 antibody
	APC labeled Rat Anti-Mouse IL-4 antibody
	PE labeled Rat Anti-Mouse IL-17A antibody FITC labeled Rat Anti-Mouse IFNγ antibody
Cocktail 2 (C2)	PerCP-Cy™5.5 labeled Rat Anti-Mouse CD4 antibody
	PE labeled Rat Anti-Mouse CD8b.2 antibody
	FITC labeled Rat Anti-Mouse IFNγ antibody

## Data Availability

Authors can provide data if requested.

## Supplementary Materials

The following are available online at https://www.mdpi.com/article/10.3390/vaccines9050507/s1, Figure S1: Representative flow cytometric analysis for splenocytes alone stained for IL-4, IFNγ and IL-17, Figure S2: Representative flow cytometric analysis for splenocytes stimulated with OVA or mock-stimulated and stained for IL-4, IFNγ and IL-17.
