# Supplementary material for: The Adjuvants Polyphosphazene (PCEP) and a Combination of Curdlan Plus Leptin Promote a Th17-Type Immune Response to an Intramuscular Vaccine in Mice
Source: Vaccines (Basel). 2021 May 14;9(5):507. doi: 10.3390/vaccines9050507 (PMC8156850; doi:10.3390/vaccines9050507)
Supplement: Supplementary file 1 [file vaccines-09-00507-s001.zip › vaccines-1131938-supplementary.pdf]

Supplementary Figures:

Supplementary Figure S1:

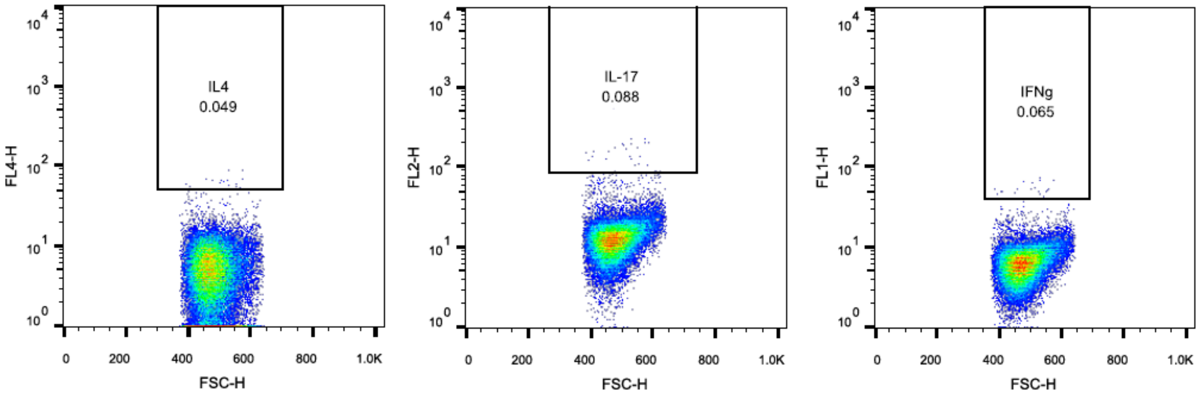

Supplementary Figure 1: Representative flow cytometric analysis for splenocytes alone stained for IL-4, IFNγ and IL-17. Gating was set based on a cells-alone stain for each animal to ensure auto-fluorescence was not detected, additionally, isotype control stains and FMO stains were due to specific antibody binding of the anti-IL4 (right side), anti-IL-17 (center), and anti-IFNγ (left side) antibodies. Antibodies were previously validated on mouse targets.

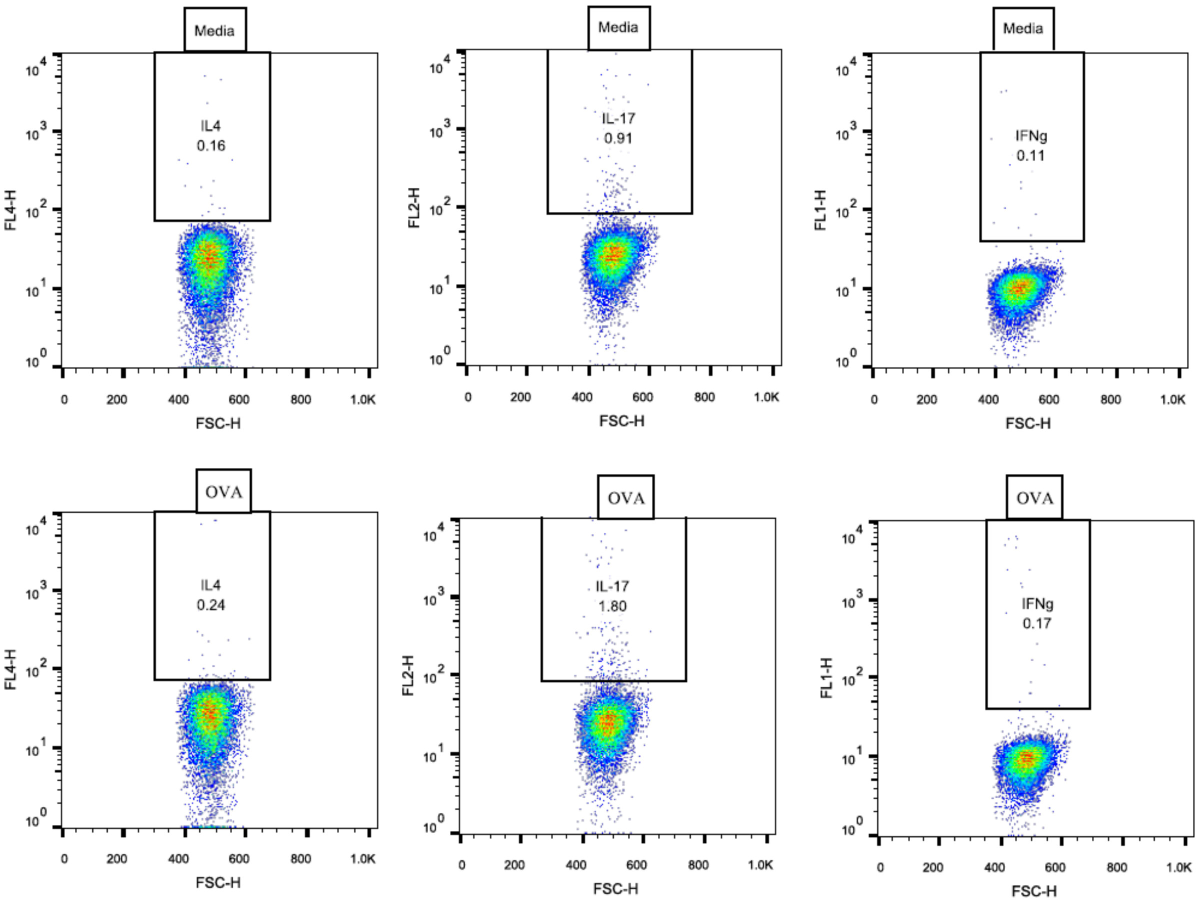

Supplementary Figure S2: Representative flow cytometric analysis for splenocytes stimulated with OVA or mock-stimulated and stained for IL-4, IFNγ and IL-17. Gating was set based on a cells-alone stain for each animal to ensure auto-fluorescence was not detected, additionally, isotype control stains and FMO stains were carried out to ensure positive events were due to specific antibody binding of the anti-IL4 (right side), anti-IL-17 (center), and anti-IFNγ (left side) antibodies. Antibodies were previously validated on mouse targets.
